# Case of Apoplexy

**Published:** 1811-09

**Authors:** 

**Affiliations:** Physician to his Majesty's Fleet, Leeward Islands


*For the Medical and Physical Journal.*

*Case of Apoplexy; by* DR. DICKSON, Physician to his Majesty's Fleet, Leeward Islands.

ON the 1st of December, 1802, I was requested to visit a merchant of the first respectability, at one of our factories, whose life was considered in such imminent danger, as to be altogether despaired of by his medical attendants.

On seeing the patient I certainly concurred with them in opinion, that a few hours would terminate his existence: there was a total suspension of all sensation, and voluntary motion; the breathing very laborious, slow, and sonorous; the pulse low, and irregular; the mouth open; countenance sunk, and ghastly; and the body covered with a cold clammy sweat.

He was only twenty-six years of age, married, and of an athletic form: had been accustomed to take violent exercise, in shooting, during the hottest months, which was supposed to have been the origin of a headach and giddiness, with which he was often affected. For a considerable time past, his health and spirits had much declined; and of late he had been attacked with intermittent fever. Within a few days, the headach and vertigo had become more frequent, and distressing; and on the preceding afternoon, he suddenly fell down in an apoplectic fit.

I may here remark that while I was receiving this account, his friends, anxious that he should have the benefit of every assistance, had sent for a Jew who professed some knowledge of medicine, who advised the application of leeches around the anus. As I was unacquainted with his reason, and did not then know that the patient had been accustomed to the hæmorrhoidal discharge, which lately had not returned as usual, I could not forbear smiling at the apparent futility of this proposition, which, in consequence met with no farther attention.

Upon inquiry I found he had been treated according to the inert and exploded doctrines which influenced the practice in the East. Small blisters had been applied to the inside of the thighs, legs, and to the nape of the neck; but to my astonishment nishment and regret, not to the head: Pigeons, just killed and opened, were applied from time to time to the soles of the feet, and a mixture, denominated cordial, had been attempted to be given, which appeared to be little stronger than liquorice water.

Finding that he was unable to swallow, the only chance in such extreme depression of the animal powers, seemed to depend upon their excitement by the application of stimulants to the surface and intestines. A large blister was, therefore, immediately applied all over the head; acrid sinapisms of mustard, horse-radish, sal ammoniac, and vinegar to the feet, and a cathartic injection was recommended to be administered. In the event of his being able to swallow, a cordial mixture was also prescribed, but very little of it, or none at all could be got down.

During the night the breathing became less stertorous, and the perspirations less profuse, and he groaned occasionally, and moved his feet as if he felt pain from the sinapisms which were directed to be renewed.

In the morning the lethargic symptoms were materially diminished, and the pulse was more free and fuller. He opened his eyes occasionally, and looked around; swallowed what was offered him, though with some difficulty, and seemed to understand what was said, but could not articulate so distinctly as to be understood. The sinapisms had excited redness, but as the vesications on the head were only partial, and the bowels remained torpid, a fresh blister was applied to the scalp, and a stimulating injection administered.

On calling in the afternoon, I was told he had shewn much inclination to sleep, and found the stupor and all the symptoms of sensorial oppression increased. As I now conceived it of the utmost importance to rouze the action of the intestines, an acrid clyster was prescribed, composed of sulphate of magnesia in an infusion of senna, with some compound extract of colocynth, and antimonial wine, which after being retained for some time, produced two scanty dejections, followed by a considerable quantity of very thick, dark-coloured blood. Similar evacuations of blood and gelatinous matter recurred repeatedly during the night, with frequent groaning as if he were much griped; and as from the appearance of the discharge, the increase of sensibility, and of the pulse, I now entertained more sanguine hopes, nothing was done to counteract the irritation excited by the clyster, except the application of fomentations to gratify his friends, who judging by his moaning and uneasiness that he was still worse, now imagined that any further proceedure ceedure would only tend, unavailingly, to increase his sufferings.

On the morning of the 3d, he was free from coma and perfectly sensible: he spoke intelligibly, and took some salop with wine. The purging recurred at intervals throughout the day, mixed with a little blood and gelatinous matter; and, as he complained of griping and tenesmus, emollient injections were now permitted, consisting of a decoction of linseed and marsh-mallow, with gum arabic, and the yolks of eggs.

From this period he recovered rapidly, and his strength and appetite improved daily: he was recommended to take tonics, and particularly to keep his bowels very open; and on the 15th, when I left him, he was nearly well and able to attend to his affairs.

How far the suppression and congestion of a discharge like the hœmorrhoidal become constitutional, might contribute to the production of the disease in question, I shall not take upon me to say; but the appearance of the blood, and the instant amendment that followed its removal, are favourable to this idea, and point out the propriety of inquiring into the patient's history, and whatever can throw light on the source of the disease, with a view both to its prevention and cure.

This case is only intended to illustrate the benefit resulting from the exciting and evacuating powers of irritating clysters in comatose affections, of which I have since seen several instances: indeed it is unnecessary to remark, that these and blisters constitute the principal resource, under circumstances where venæsection is improper, and purgative, or other remedies by the mouth cannot be exhibited.

